# A feeling of flow: exploring junior scientists’ experiences with dictation of scientific articles

**DOI:** 10.1186/1472-6920-13-106

**Published:** 2013-08-10

**Authors:** Lene Spanager, Anne Kjaergaard Danielsen, Hans-Christian Pommergaard, Jakob Burcharth, Jacob Rosenberg

**Affiliations:** 1 Danish Institute for Medical Simulation, Capital Region of Denmark, Herlev, Denmark; 2 Department of Surgery, Herlev Hospital, University of Copenhagen, Copenhagen, Denmark

**Keywords:** Education, Dictation, Scientific manuscript, Flow, Writing team

## Abstract

**Background:**

Science involves publishing results, but many scientists do not master this. We introduced dictation as a method of producing a manuscript draft, participating in writing teams and attending a writing retreat to junior scientists in our department. This study aimed to explore the scientists’ experiences with this process.

**Methods:**

Four focus group interviews were conducted and comprised all participating scientists (n = 14). Each transcript was transcribed verbatim and coded independently by two interviewers. The coding structure was discussed until consensus and from this the emergent themes were identified.

**Results:**

Participants were 7 PhD students, 5 scholarship students and 2 clinical research nurses. Three main themes were identified: ‘Preparing and then letting go’ indicated that dictating worked best when properly prepared. ‘The big dictation machine’ described benefits of writing teams when junior scientists got feedback on both content and structure of their papers. ‘Barriers to and drivers for participation’ described flow-like states that participants experienced during the dictation.

**Conclusions:**

Motivation and a high level of preparation were pivotal to be able to dictate a full article in one day. The descriptions of flow-like states seemed analogous to the theoretical model of flow which is interesting, as flow is usually deemed a state reserved to skilled experts. Our findings suggest that other academic groups might benefit from using the concept including dictation of manuscripts to encourage participants’ confidence in their writing skills.

## Background

Writing and publishing are critical to academic success. Some academics master these tasks, many do not. Commonly cited barriers to publishing are lack of momentum, lack of available time, and lack of confidence in writing [1]. This is often referred to as “writer’s block”, meaning that it is difficult to get started and produce the first manuscript draft [2]. Different initiatives have been tested to help academics overcome writer’s block including peer writing teams [2,3], writing retreats [4], and combinations of the two [5-7]. All these initiatives have proved effective in raising the publication rate of the participants.

Very little literature describes scientists’ experiences with dictation of scientific articles. The few reports of dictation being less time consuming than writing [8] and easy to learn [9] covers letter writing and come from the 1970s, before the widespread use of computers. More recent studies have suggested that dictation combined with a manuscript outline is more effective than writing with pen and paper for children and students with a learning disability [10]. To our knowledge, studies on the effects of dictation for scientists are lacking.

*Writing for publication* was an educational framework initiated for junior scientists with the aim of increasing publication rates. The key component was using dictation as a method of producing a first manuscript draft and it was supported by writing teams and dedicated time for dictation during writing retreats [11]. *Writing for publication* was initiated with an opening 2-hour kick-off seminar covering topics such as explanations of article composition, guidance on how to write a structured manuscript outline, and instructions for dictating. Subsequently, the framework centered around three separate courses over 10 months. Each course comprised three phases: a 4-week preparation period for creating a structured manuscript outline, a one-day retreat for dictating a scientific manuscript draft, and a subsequent 8-week period for critically revising the manuscript. Supervisor-led writing teams supported all three phases. Participants were told to expect that dictating would make the writing process smoother, creating a text with a linguistic level between spoken and overtly scientific language. By the end of the courses each participant had produced (dictated) three articles that were submitted to peer-reviewed scientific journals within a few months of the 10-month study period, so the framework was effective in terms of ensuring high submission rates. However, how the junior scientists responded to the dictation method remains unexplored. We therefore chose an inductive approach, using interviews to answer the research question: what characterized junior scientists’ experiences with the process of dictating scientific articles, and their attitudes towards participating in writing teams and writing retreats within the *Writing for publication* framework?

## Methods

### Setting

The educational framework was initiated at a university hospital in Denmark in September 2011. All participating junior scientists were affiliated with the same clinical professor who introduced the technique and initiated the framework. The scientists were PhD students and scholarship students, i.e. in their junior research years with limited publication experience. All participants were native Danish-speakers. The Danish pre-graduate medical education gives very limited research experience, so some medical students take a year’s leave to do research (i.e. a scholarship year), and the current PhD program at the Faculty of Health Sciences does not include training of writing skills with the use of dictation. Therefore, the framework was developed to address the need for structured and guided training of writing skills with a simple technique to increase publication rates while preserving good quality of the produced papers [11].

### Design

The study draws on a qualitative study design using focus group interviews [12] and was conducted October 2011–January 2012. Interviews were set-up with a descriptive phenomenological approach to grasp the central understanding of the participants [13,14] and to create discussions among them. We deemed that the use of focus groups would enhance the descriptive phenomenological approach [15], rather than considering the two incompatible [16]. We used purposive sampling [17], including all participants in the first of the three *Writing for publication* courses.

### Interviews

The interview guide was semi-structured and consisted of open-ended questions (see the list of Saints section). It was constructed to explore five phases of the *Writing for publication* course, covering: idea, manuscript outline, dictation, revision of manuscript, and submission of the article. The interview questions were designed to elicit what participants thought worked well and what did not work during the five phases and to explore the reason for it. The interview guide also contained questions regarding the role of supervisors, writing teams and writing retreats.

### Interview guide

1. Please describe your experiences with and attitudes towards:

a. The idea phase

b. The creation of a manuscript outline

c. The process of dictating

d. Revision of the manuscript

e. Submission of the article

2. What worked and what did not work? Please describe why and how

3. What were your experiences with the supporting functions of the *Writing for publication* framework?

a. Academic advisors

b. Writing teams

c. Co-authors

4. If you dictated in a foreign language (English) then what were challenges? And advantages?

5. Comments regarding type of article dictated?

6. Anything else you would like to add?

Interviews were conducted in four focus groups. Together, the first three groups comprised all participants who had recently attended the first course. The fourth group comprised all participants and was a follow-up interview a few months later aimed to explore experiences of the review process; accordingly, all participants were interviewed twice. Interviews were conducted by two experienced interviewers (L.S and A.K.D), who participated in the *Writing for publication* framework but who were not interviewees. Interviews lasted 51–111 minutes (range), were digitally recorded and transcribed verbatim.

### Analysis

We aimed for a level of analysis that would provide descriptions of the informants’ experiences [18]. Qualitative content analyses of emergent themes were applied [19] and the unit of analysis was each interview. The two interviewers independently read each transcript and coded it by first identifying meaningful units of text, then condensing the meaningful units to the essential content, and finally coding the meaningful units. Subsequently, the interviewers discussed each coded interview transcript face-to-face and agreed on a coding structure, which was applied to the rest of the transcripts, ultimately clustering the codes into categories and subsequent themes. A few meaningful units were sorted under two themes.

### Ethical considerations

Written informed consent was obtained from all participants. They were informed that they were able to withdraw from the study at any time. Data were anonymized when transcribed and analyzed. The regional ethics committee was contacted and stated that the study was exempt from ethical approval as it was not by definition a biomedical research study (Journal number: H-2-2013-FSP49). Registration at clinicaltrials.gov was not applicable considering the nature of the study.

## Results

The participants were 7 PhD students, 5 scholarship students and 2 clinical research nurses with a median age of 30 years (range 25–49). They produced manuscript drafts for a range of article types including original articles, narrative and systematic reviews, and case reports, and they all dictated a full manuscript draft in one single session lasting 1.5–7.0 hours. Their scientific writing experience comprised a median of 4 (range 0–15) articles submitted to peer-reviewed scientific journals and most dictated a scientific article for the first time during the first course.

We identified three main themes in the interviews. The themes and subthemes are described in detail in the following and the Table 1 gives quotations that underpin the descriptions.

**Table 1:** Themes, subthemes and quotations from interviews

**Theme**	**Subtheme and quotations**
Preparing and then letting go	**Preparation**
	‘I agree the work has to be done. The question is whether you want to put the effort in to the manuscript outline or struggle with it afterwards. There is no way out of critically deciding what has to go in the article.’ (Male, interview 4)
	**Dictation**
	‘I think that to be able to dictate… I mean the entire process of dictation requires that you release control in relation to perfectionism. At least I experienced that sitting there with this Dictaphone, and for me it was the first time and it was really challenging to let go of all these words. As opposed to the control associated with a document, […] so to express oneself to this machine was atypical and unusual.’ (Female, interview 4)
	**Revision**
	‘My first draft was a FIRST draft. I could see that it was not entirely coherent and the language was not short […] Apparently I can talk for a very long time without dots.’ (Female, interview 4)
The big dictation machine	**Writing teams**
	’Well, I don’t know if you could use any of the feedback we gave you, but we all contributed to your subject without grasping all the details of it. It was not just the team leader that gave good advice. The statistics were also discussed in the team, so I see a clear advantage in trying to understand each other’s subjects and using it to learn something yourself’ (Female, interview 1)
	**Time pressure**
	’I also think that we had too little time from starting until we had to go [to the retreat]. I would have liked more time; I did not achieve all I wanted in the preparatory phase. (Female, interview 2)
	**Undisturbed surroundings**
	‘I think there is the advantage in the feeling of *community* that others are sitting in the room next door dictating. On the other hand it is also a bit stressful when you sense doors opening and people are finishing, and I haven’t finished yet […] But the advantages that out-weighs everything is that others are close by doing the same thing – it’s like: Wow we’re being productive and it will be good to celebrate with the others this evening. You look forward to this as opposed to just going home, it wouldn’t have been the same feeling. A feeling that helps with the process of dictating.’ (Female, interview 4)
	‘Well the social aspect goes hand in hand with working and that was the idea. You feel like a big article-writing machine’ (Male, interview 4)
Barriers to and drivers for publication	**Motivation**
	‘It may be connected to the fact that it was a subject that came upon because we were going to the retreat. And then it became, well something that just had to be done so I could get back to what I am really working on right now. It is possible that my feeling would be different if the subject was something I was going to focus on for a while.’ (Female, interview 1)
	’It is more fun to work on a subject that you have chosen yourself, that you have helped develop and something you find interesting. But especially on this occasion – with this type of paper that was different from the sort I usually write, since it was an editorial, and within my field of research in which I am still excited that we have found something new – that was in fact motivating in itself.’ (Male, interview 3)
	**Flow**
	’I still really can’t put it into words, but for me the flow process is synonymous with momentum. When you get into a phase of productivity where 2 plus 2 equals 4, because you get more done, you can do more, I think faster and work more efficiently. And it is on the other hand, like X said, dreadful when you for some reason don’t have it – due to busy times or other activities. It is really a feeling to make you feel high; when you make something you succeed with, fast and efficiently and you get it submitted. It is the most awesome feeling and on the other hand it is stressful not to feel it, once you have tried.’ (Male, interview 4)
	’I think that for me it [dictating] is absolutely fantastic. It is perfect for my way of thinking. I do not think that my written language differs much from my spoken language. And it sets the creative process free, because you can associate much faster. And while you are in the middle of constructing a sentence, then something new pops up, something that you hadn’t thought about and you can dictate that right away. You get deeper into a subject. I feel that there is no brake - that the language flows better when I dictate, than when I write.’ (Male, interview 2)

### Preparing and then letting go

#### Preparation

The production of a manuscript included a preparation phase. For those who had sufficient time and who were familiar with the content and scope of their article, this functioned optimally and saved time in the revision phase. Several participants experienced that their manuscript outline needed several revisions, including immediately prior to dictating at the retreat. Getting the outline “out of one’s head and down on paper” was described as positive, just as working with an outline helped to remain focused and to stick to the planned message of the article.

#### Dictation

Dictation started hesitantly but became easier during the process. It involved letting go of control, but was then rewarded with a sense of immediate liberation. Usually this occurred during the discussion section of the article. Most informants found it faster to dictate than to write, and for some it was easier to communicate orally than “through their fingers”. One described that she missed seeing the written words and it was discussed how dictation in a foreign language (English) was more difficult. It usually took time to find the English words; however, this was beneficial as the sentences naturally became shorter.

Some experienced the liberation as purely enjoyable. Others simultaneously appreciated it and marveled at the indifference they felt regarding the linguistic quality and coherence of the manuscript, as long as the content was complete. Almost everybody found it difficult to remember what they had only recently said. It puzzled some, while others considered it a sign of focus and used the manuscript outline to follow their progression. Some did not experience the language fluency that they had expected.

#### Revision

The first draft showed that the process of dictation was “quick and dirty”, meaning that the overall structure of the manuscript was in place, but for some the manuscript required several feedback sessions with the supervisor before being ready for submission. In addition, the draft revealed whether the preparation had been good or poor. Many described good experiences in keeping momentum by revising the draft immediately after the dictation, contrasting the frustration described by others when they were unable to do the revision immediately. Nevertheless, the relative emotional detachment that occurred for those who had to delay the revision made it easier to delete redundant paragraphs and sentences.

### The big dictation machine

#### Writing teams

Writing teams functioned well when the framework and meeting frequency were quickly settled. It was considered helpful when milestones were outlined and monitored. Writing teams ensured a high level of preparation and progression, especially when team members received guidance on the content of the paper and not merely on manuscript structure. Some mentioned a very positive social aspect of participating in writing teams as it enhanced collaboration beyond the framework. Some were clearly able to give feedback on others’ work, whereas others felt insufficiently skilled to do this. It was recognized that active participation from all team members was a prerequisite for optimal team functioning, and that guidance was optimal if the team leader was also the general supervisor of the project and hence familiar with the content.

#### Time pressure

Everybody experienced a certain time pressure during the process. That often led to a curtailed literature search or an inadequately prepared manuscript outline. Some even had to collect and analyse data concurrently with the creation of the manuscript outline. All were able to handle the time pressure though, and a few even considered the external pressure rewarding.

#### Undisturbed surroundings

Being physically away from ones normal workplace during the dictation process made it impossible to deviate from the core work process, and the writing retreat gave a feeling of community that was considered cozy, inspirational and very productive at the same time. The disadvantage was that some experienced a pressure to finish the dictation process quickly, as a certain competition arose among participants.

### Barriers to and drivers for publication

#### Motivation

Motivation was crucial for a feeling of success as the process had to have a purpose. The article either had to be relevant for the participant’s research project, or others’ subsequent work should depend on it. For some it was a good opportunity to finish a draft for an outline that had been put aside. Participants who had conceived the idea for the paper themselves described more motivation and a greater feeling of ownership of the project than did those who had the topic presented to them. Clearly, it was much easier to work with a self-chosen topic than writing something obligatory.

#### Flow

A flow-like feeling occurred for many of the scientists during the dictation process, half of them talked about it in various ways. For some the descriptions included a fantastic perception of rapid association and full overview of the subject while dictating, although the process was associated with a tendency to forget what they had just said. Others described a feeling of great satisfaction from finishing a complicated task in just one day. It provided informants with energy and reasons to want to experience this again. One of the scientists, who had tried dictation of a scientific article more than once, elaborated that the flow-like feeling was volatile, i.e. not present every time. Motivation played a crucial part in gaining the feeling of flow, but motivation in itself was no guarantor for a flow-like experience. Flow was almost addictive; doing without it once it had been experienced was hard. Participants discussed whether flow could be experienced at any time during the process and most of them who had experienced it found that they felt flow during dictation of the more loosely structured discussion part of the manuscript.

## Discussion

This interview study exploring participants’ experiences relating to dictating scientific articles for the first time, participating in writing teams and attending a writing retreat, showed that dictation worked best when properly supported and when participants were motivated. When this was the case, some had experiences that resembled a feeling of flow. Dictation was successful for all participants, but it required a high level of preparation.

For some it was difficult to prepare for the retreat, have the data analysed and the literature search ready before drafting the outline of the manuscript. This was probably because of the time pressure in the four-week preparation period. Creating a manuscript outline has been recognized as a means of improving text quality and text fluency in written manuscripts [20]. A high level of preparation is most likely pivotal in being able to dictate a full article in one day. Writing is a process that connects to learning processes and memory optimizing cognitive skills [21]. The same probably also applies to dictation, which might be considered a process to be learned in the same way that writing is.

Others have shown that writing courses can improve publication rates, particularly when combined with writing teams [5-7] and that participating in writing teams can increase novices’ confidence in their own writing skills [6]. People learn not only through their own experiences, but also by observing and talking to others through collaborative learning processes [22,23]. Although having regular, structured meetings with participants and receiving encouragement from peers were previously reported as effective aspects of writing teams [2], the study also reported that not all writers were comfortable receiving criticism on their manuscripts from peers [2]. This was not mentioned in our interviews with junior scientific writers. By contrast, they appreciated the feedback they received, but it seemed to be a barrier to some that they were expected to provide feedback for others when they felt incompetent to do so. Another challenge regarding writing teams and attending a retreat was the element of competition that seemed to arise when some of the participants finished the dictation process sooner than others. Both of the above drawbacks could be a result of our young study population. Another important downside was related to situations where the team leader provided guidance only on the process and not on the content of the paper.

Flow and a flow-like sensation were frequently mentioned throughout the interviews and were tightly connected to feelings of joy and motivation. The descriptions of flow-like mental states seem analogous to the theoretical model of flow [24] as a psychological state in which the person feels simultaneously cognitively efficient, motivated and happy [25]. The participants’ descriptions were on a common sense level but were in line with flow described as occurring when a person experiences an optimal balance between his/her skill level and challenge level; keeping both boredom and anxiety at a distance. We consider this an interesting finding, as flow is usually deemed a state reserved to very skilled experts. In this study, novices described that their feeling of a flow-like state *per se* was a motivational driver to do the job and do the job once again.

Motivation was also frequently discussed in the interviews as key to the good outcomes of the framework. Motivation can be internally or externally driven. This study’s sample of PhD students, scholars and clinical research nurses was likely highly internally motivated. So our results match what other interventions have shown for scientists with few publications, which are little confidence in writing skills and a desire to improve writing skills [2]. In this *Writing for publication* framework it was repeatedly and explicitly mentioned that the aim of the intervention was to overcome writers’ block and thereby increase publication rates. Consequently, participants might have felt a social expectation of participation in the program and to be productive during this, thereby contributing with externally driven motivation. It is also a possibility that this external pressure to write for publication helped stimulate the participants’ arousal level, thereby pushing them towards a state of flow [26]. Future studies could explore the relationship between dictation and flow-like feelings further, preferably with scientists not associated with the *Writing for publication* framework to test whether the feeling arises due to the dictation process or the external circumstances.

This study has limitations. Being a qualitative study, the possibilities of generalization are limited, and any referral of findings should be related to situations and participants similar to the ones in our study [27]. Firstly, it draws on a small sample size of only 14 participants. This resulted in four interviews in which only the last focused on the revision phase of the manuscript drafting. However, saturation was achieved regarding participants’ experiences relating to the preparation, dictation and revision of a manuscript. The two interviewers were also participants in the course, potentially influencing informants and biasing the analysis. However, during the interview process and the subsequent analytical process, both researchers kept a constant focus on this issue to prevent any methodological flaws. The comparative method applied in the analytical phase ensured that inferences were based on the transcripts to sustain the connection between data and final findings. Finally, study participants were all from the medical field (students, doctors and nurses), and all from the same hospital.

## Conclusions

For many inexperienced scientists this framework of *Writing for publication* including participation in writing teams and dictation of an article at a writing retreat was a successful experience. We found motivation and proper preparation to be crucial factors leading to the success, and that novices experienced flow-like states of mind. This suggests that other academic groups could benefit from using the concept including dictation of first drafts of manuscripts to encourage participants’ confidence in writing skills and raise publication rates.

## Competing interests

The authors report no declarations of interest.

## Authors’ contributions

LS, AKD and JR conceived the idea. LS and AKD designed the study and the interview guide, conducted the interviews, coded and analysed all the transcripts. JR, HCP and JB contributed to data interpretation. LS drafted the manuscript with help from AKD. All authors critically revised the manuscript and approved the final manuscript.

## Pre-publication history

The pre-publication history for this paper can be accessed here:

http://www.biomedcentral.com/1472-6920/13/106/prepub
